# Multipoint Lock-in Detection for Diamond Nitrogen-Vacancy Magnetometry Using DDS-Based Frequency-Shift Keying

**DOI:** 10.3390/mi15010014

**Published:** 2023-12-21

**Authors:** Qidi Hu, Luheng Cheng, Yushan Liu, Xinyi Zhu, Yu Tian, Nanyang Xu

**Affiliations:** 1 Research Center for Quantum Sensing, Zhejiang Lab, Hangzhou 311000, China; 2021171407@mail.hfut.edu.cn (Q.H.); 2021111211@mail.hfut.cn (L.C.); yslw@mail.hfut.edu.cn (Y.L.); 2021111210@mail.hfut.edu.cn (X.Z.); tianyu751@163.com (Y.T.); 2 School of Microelectronics, Hefei University of Technology, Hefei 230009, China; 3 Institute of Quantum Sensing and College of Optical Science and Engineering, Zhejiang University, Hangzhou 310027, China

**Keywords:** field programmable gate array, quantum precision measurement, direct digital synthesizer, acquisition and processing equipment, microwave source

## Abstract

In recent years, the nitrogen-vacancy (NV) center in diamonds has been demonstrated to be a high-performance multiphysics sensor, where a lock-in amplifier (LIA) is often adopted to monitor photoluminescence changes around the resonance. It is rather complex when multiple resonant points are utilized to realize a vector or temperature-magnetic joint sensing. In this article, we present a novel scheme to realize multipoint lock-in detection with only a single-channel device. This method is based on a direct digital synthesizer (DDS) and frequency-shift keying (FSK) technique, which is capable of freely hopping frequencies with a maximum of 1.4 GHz bandwidth and encoding an unlimited number of resonant points during the sensing process. We demonstrate this method in experiments and show it would be generally useful in quantum multi-frequency excitation applications, especially in the portable and highly mobile cases.

## 1. Introduction

NV centers are atomic-scale defects in diamond and are an important component of quantum technologies, including quantum sensing [1], quantum computing [2,3,4] and quantum simulation [5,6]. NV has been proved to be an excellent sensor, which can detect magnetic field [7,8,9,10,11], electric field [12,13], temperature [14,15], and pressure [16], etc. Due to its characteristics of a short polarization time and long coherence time, NV centers have become a research highlight in recent years. Through the continuous wave optically detected magnetic resonance (cw-ODMR) experiment [17], we can easily detect the NV centers in diamonds which require a microwave (MW) source with high resolution and a data acquisition (DAQ) module whose role is processing fluorescence data collected by photodetectors (PDs).

Through traditional lock-in methods, we can measure fluctuations in temperature and maganetic detection with single resonance [18] or multiple resonance [19]. When extending a lock-in method to multipoint measurement, previous experiments presented two feasible solutions. The first involved using multiple MW sources, but this approach was found to be unfavorable for subsequent integration and miniaturization within small-scale integrated systems. Additionally, due to the need to avoid intermodulation interference, the entire system was designed to be highly complex. The second option involves switching the microwave after detecting a signal resonance peak, but this may result in reduced data timeliness.

In this article, we report a multipoint lock-in detection method applying a self-built MW source and sampling module. The architecture of the source is based on DDS and FSK. With the use of DDS, the minimal time required for frequency shifting can reach sub- μ s level and its maximum bandwidth is 1.4 GHz. In order to obtain a cleaner signal, we control its output more than 500 MHz in practical use. FSK ensures arbitrary frequency hopping of the MW source and synchronization with the sampling module. Sampling module can realize collaborative work with the MW source through a trigger signal. The collected fluorescence signal will be encoded according to the current frequency generated by the source and transmitted to the PC through Ethernet. Finally, the encoded data will be analyzed by PC. We have applied the system in cw-ODMR and multipoint lock-in detection, and thus demonstrate its performance. The method reported in this article can be used in practical applications such as miniaturized detection equipment due to its integrated architecture. Because of its ability to measure multiple resonances, we can employ it to observe temperature and magnetic field fluctuation in the environment.

## 2. Implementation

**Figure 1:**
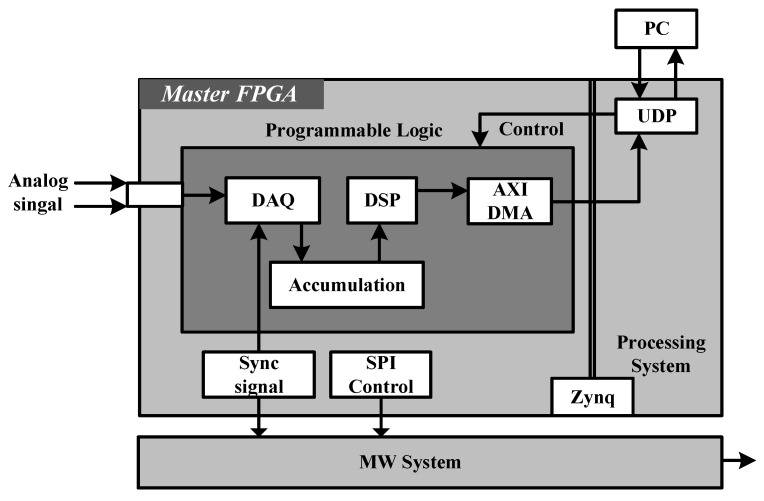
The overall structure of the digital system we reported. The master FPGA (Red Pitaya) is responsible for receiving orders from PC and control MW source and sampling module (including DAQ and DSP). Except from 3-pin serial peripheral interface (SPI) [26], the master–slave FPGA is connected by a trigger signal, which could ensure the signal synchronization between signal sampling and microwave generation.

The overall structure of digital systems we reported is shown in Figure 1. From the perspective of hardware, the system can be mainly divided into the following two parts. The first part is a master field programmable gate array (FPGA) whose primary component contains an FPGA with processer (ARM Cortex A9), a core (Xilinx Zynq XC7Z010) [20] chip, 14-bit dual ADC (LTC2145CUP-14) with a sampling rate up to 125 MSPS and DAC (AD9767) chips. And the second part is a self-built microwave source based on direct digital frequency synthesis (DDS) [21,22,23], and all the components are integrated on a 60 mm × 50 mm single printed circuit board (PCB), including a DDS chip (AD9914), a slave FPGA (Xilinx Artix-7) [24], two wide band phase-locked loops (PLLs) [25], a frequency mixer, power amplifier system, and several frequency filters. From the perspective of function, the system can be divided into following parts: DAQ is meant to sample the analog signal collected by PDs, the digital signal processor (DSP) is used to encode and process the data, and the embedded system forms a connecting link between the hardware, PC host and sampling module. Finally, PC is the terminal of the whole system and is responsible for analyzing data.

### 2.1. Microwave Source

**Figure 2:**
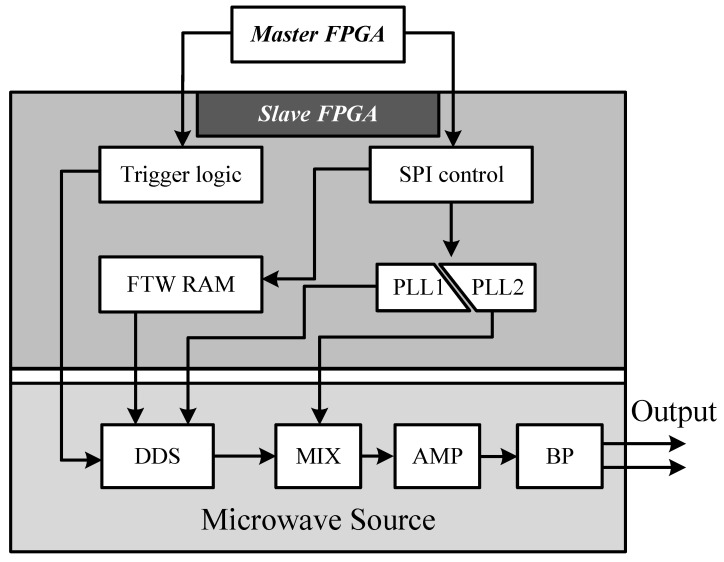
The architecture of MW source. The slave FPGA receiving orders from the master FPGA through SPI and the MW sourece is synchronized with DAQ and DSP with a trigger signal. Two pieces of PLL are used in the MW source, PLL1 generates 3.5 GHz for DDS’s reference clock, and PLL2 is configured to output 3.5 GHz in our experiments, which use mixer’s LO signal. Therefore, using down-conversion, we obtain a microwave signal from 2.5 GHz to 3.0 GHz. Additionally, the MIX and AMP module in the figure represents a mixer and amplifier which are applied to adjust the frequency and power of the microwave. Finally, the microwave will pass through a bandpass (BP) filter to make the signal clean.

The architecture of MW source is shown in Figure 2. Initially, the master FPGA receives FTWs from the PC, converts them into binary numbers, and transmits them to the slave FPGA via the SPI interface. Upon receiving the data, the slave FPGA stores the FTWs in RAM, while the DDS enters a preparation state, awaiting a trigger signal. When the instruction to start the micreowave is issued by the PC, the master FPGA initiates the transmission of a trigger signal, which is a square wave with an adjustable duty ratio. Finally, the DDS generates the microwave based on the FTWs, and the frequency and power of the microwave are adjusted to meet the experimental requirements using mixers and amplifiers [27].

In order to manipulate the spin of qubits in NV centers, it is essential to have a continuous wave that corresponds to the magnetic resonance. The spectrum of NV centers exhibits four groups of resonances due to their structure. To detect different resonances, our reported MW source offers both scanning capabilities across the spectrum and the ability to hop between custom frequency points. This functionality is achieved through the utilization of frequency turning words (FTWs) stored in random access memory (RAM).

Note that in addition to the FTWs, a frequency offset word (FOW) is stored in the RAM and it is applied for multipoint lock-in detection; the reason why we need this will be explained in the experimental section. The conversion relationship between FTWs and microwaves’ frequencies can be configured using the following equation:

(1) $$\mathrm{FTW}=\mathrm{round}(\frac{f_{out}}{f_{ref}}\cdot 2^{32}).$$

In this equation, when only FTW is utilized to control the DDS, $f_{out}$ denotes the final output of the DDS and the variable $f_{ref}$ represents the reference signal generated by PLL with a bandwidth ranging from 23.5 MHz to 6 GHz, and it is configured to be 3.5 GHz according to experimental requirements. When FOW is added, the equation turns into:

(2) $$\mathrm{FTW}\pm \mathrm{FOW}=\mathrm{round}(\frac{f_{out}^{\prime }\pm f_{offset}}{f_{ref}}\cdot 2^{32}).$$

**Figure 3:**
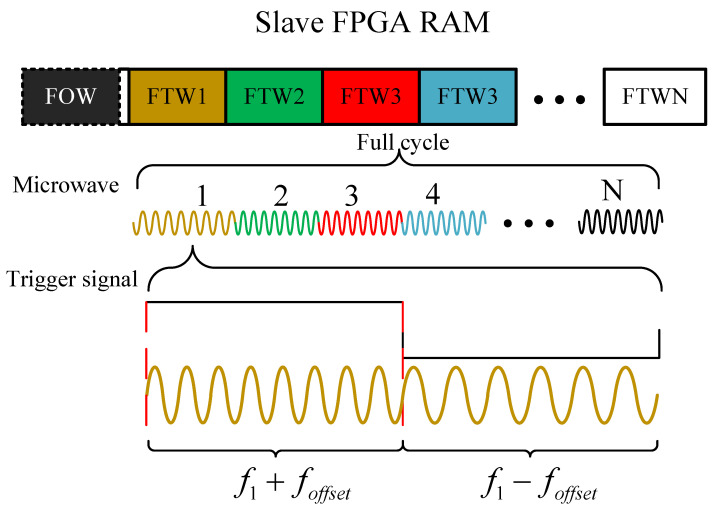
The signal adopts FSK modulation, where the microwave frequency changes based on the rising and falling edges within a single cycle, while the center frequency of each cycle varies according to the FTWs.

In Equation (2), $f_{offset}$ represents the frequencies to perform FSK modulation, and we aim to set it at a lower value to acquire data resembling a differential signal. $f_{out}^{\prime }$ represents a frequency component determined by FTW. $f_{out}^{\prime }\pm f_{offset}$ indicates the final output frequency of DDS. FTWs and FOW are stored in the RAM of the slave FPGA. Whenever the positive edge or negative edge of the trigger signal arrives, a pointer loops through the FTW. We have developed two working methods by adjusting FTWs and FOW. When FOW is set to zero and the mappings of FTWs are evenly arranged on the spectrum, the MW source operates in scanning mode, and we apply it to cw-ODMR. It can be inferred that if the mappings of FTWs are arranged irregularly, the frequency of the output signal will hop across the spectrum. At the same time, FOW is configured to make the microwave’s frequency vary depending on the level of the trigger signal. The specific situation is shown in the Figure 3.

### 2.2. Signal Sampling and Processing

**Figure 4:**
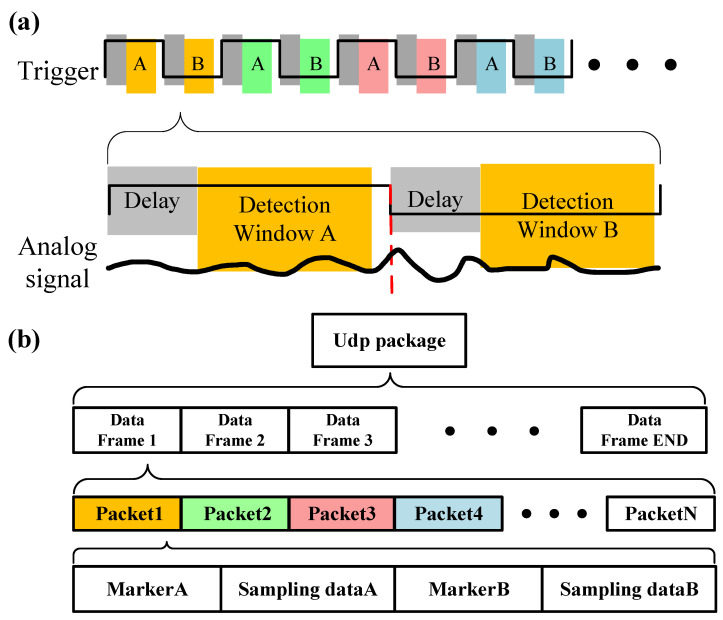
The architecture of sampling module. (**a**) Time sequence of sampling module. (**b**) After the sampling is completed, the system will package the data.

In the digital system used for multipoint lock-in detection, the frequency of the output microwave hops across the spectrum. If we simply sample the analog signal collected by the photodetector without associating it with the microwave, the data will be meaningless. Therefore, it is necessary to synchronize the microwave source, DAQ, and digital signal processing (DSP). The timing sequence of the acquisition process is shown in Figure 4.

The amplitude of signal collected by PD is 300 to 400 mV and the ADC integrated on master FPGA (LTC2145-14) has a full-scale range (FSR) of 1 V or 20 V, which can be switched using a jumper. Additionally, it has a resolution of 14 bit. Due to the master FPGA’s clock frequency is 125 MHz so the sampling module has a maximum sample rate of 125 MSPS. Due to the frequency of the magnetic field to be measured being within 100 Hz, to sum up, the ADC basically meets our experimental requirements.

Both the microwave and sampling processes are driven by the same trigger signal, allowing for synchronization between these modules. The sampling process can be divided into two parts within a cycle of the trigger signal. As depicted in Figure 4a, the gray segments attached to the trigger signal represent delay windows. During this period, the DAQ does not operate because the DDS requires time to shift frequency after the positive or negative edge of the trigger arrives. Thus, the signal during this period is discarded to avoid errors. Subsequently, the yellow segments represent the sampling window, during which the ADC samples the signal every 8 ns and accumulates the 14-bit data. It is important to note that the duration of the delay and the sampling window are adjustable, but their combined maximum should be less than the period of the trigger signal. Following the DAQ sampling, the DSP marks the data according to the frequency of the current microwave. In Figure 4b, the data are classified as part A and part B based on the level of the trigger (and microwaves’ frequency) and packaged into a 128-bit data packet containing two 16-bit markers and two 48-bit accumulated data.

Thus each FTW stored in RAM corresponds to a data packet. Every time the pointer traverses all FTWs and DSP produces N data packets (N represents the number of FTWs), we shall organize them into a data frame. After several cycles, data frames will be uploaded to PC through UDP. PC will divide the data into different groups based on the markers and analyze them.

### 2.3. Experiment Setup

**Figure 5:**
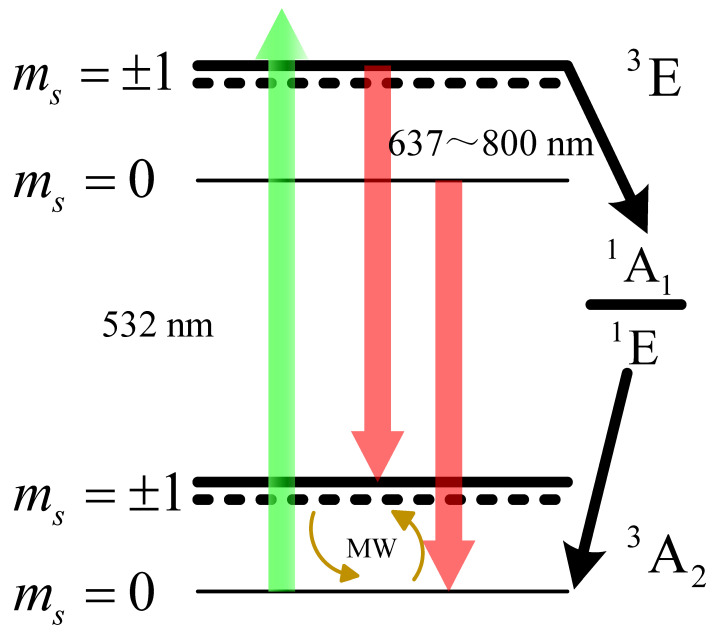
The energy level diagram of the electron spin in the NV center can be represented schematically. In this diagram, the green arrow represents the 532 nm laser, while the red arrow represents the fluorescence emitted from 637 to 800 nm. Black arrow path corresponds to the ISC transitions between the excited states $|m_{s}=\pm 1\rangle$ and ground state $|m_{s}=0\rangle$ . On the other hand, a pair of brown arrows denote the $|m_{s}=0\rangle$ ↔ $|m_{s}=\pm 1\rangle$ transitions, which can be achieved by applying resonant microwave.

The implement of experiment is based on the energy level of NV centers as shown in Figure 5. NV centers have two distinct states: $|m_{s}=\pm 1\rangle$ and $|m_{s}=0\rangle$ , representing different electron spin states. These states can be interconverted through microwave excitation, and NV centers can be excited from the ground state of ^3^A_2_ to the excited state of ^3^E. When in the state of $|m_{s}=\pm 1\rangle$ , NV centers have two routines to decay from the excited state to the ground state a few nanoseconds after they are excited with the 532 nm laser.

One route emits fluorescence from 637 to 800 nm, while the other route involves decay to the ground state through states of ^1^A_1_ and ^1^E with the assistance of intersystem crossing (ISC), which does not emit fluorescence [28,29]. Relatively, when the NV center focuses on the excited state of $|m_{s}=0\rangle$ , it can only decay to the ground state through photoluminescence. We can determine the proportion of the NV center in the $|m_{s}=0\rangle$ and $|m_{s}=\pm 1\rangle$ states based on the fluorescence intensity, as a large number of NV centers are excited by the 532 nm laser. By applying continuous microwaves whose frequency is near the resonance of one of the $|m_{s}=0\rangle$ ↔ $|m_{s}=\pm 1\rangle$ transitions on diamond with an Ω -type antenna, electronic spin can be transferred from the more fluorescent $|m_{s}=0\rangle$ ground state into the less fluorescent $|m_{s}=\pm 1\rangle$ . When the external magnetic field beside the diamond changes, the resonance of $|m_{s}=0\rangle$ ↔ $|m_{s}=\pm 1\rangle$ transitions shifts in position on the spectrum.

Mismatch between microwave and resonance would prevent most of electron spin on the state of $|m_{s}=0\rangle$ from being transferred into state of $|m_{s}=\pm 1\rangle$ , leading to enhanced emitted fluorescence. Hence, fluctuations in the external magnetic field can be traced by determining the fluorescence of diamond with NV centers. If the external magnetic field is oscillating, the fluorescence generated by the NV centers will also oscillate at the corresponding frequency near the resonance absorption peak.

## 3. Result

### 3.1. General-Purpose Experiment of NV Ensembles

**Figure 6:**
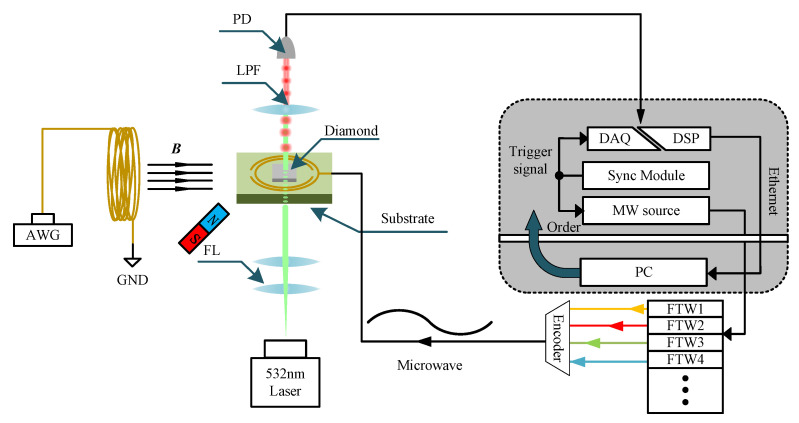
The implementation of experiment which is based on the self-built digital magnetic field detection system. FL represents focusing lens. We place a permanent magnet next to the sample to generate a static magnetic field. NV center is excited by the 532 nm laser and emitted fluorescence is collected by PD after passing through LPF (Low Pass Filter) and then translated to digital signal. Later, the digital signal will be sampled and processed by DAQ and PC decoder. Simultaneously, MW source is controlled by PC and we connect the magnetic core coil to an arbitrary waveform generator (AWG) to generate magnetic field to be measured.

To begin with, we utilize the digital system in the cw-ODMR experiment. On the one hand, this allows us to validate the performance of the MW source and DAQ with DSP. Simultaneously, we can identify the different resonances of NV centers resulting from the four different orientations of NV centers in the diamond’s crystal cell. The operation of the experiment is shown in Figure 6.

**Figure 7:**
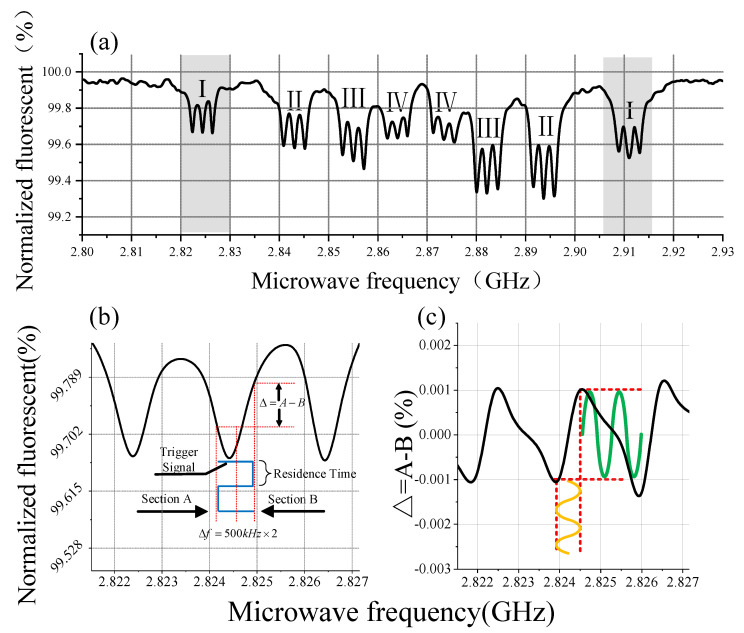
(**a**) Result of cw-ODMR, we divide resonances into four groups according to different orientations resulting from four kinds of NV centers with different orientations. (**b**) Single resonance whose frequency is near 2824.4 MHz and is marked as I in (**a**). We set the gap between section A and section B at 1 MHz = 500 kHz × 2. Δ = A − B represents the signal we obtain. The blue line represents the trigger signal. (**c**) Δ = A − B of resonance I. We obtain the oscillating fluorescence signal by differentiating the two samples which are collected with microwaves of different frequencies beside the oscillating magnetic field. In the ideal case, the yellow line indicates the sinusoidal shift of the resonance peak on the spectrum due to the external magnetic coil, and the green line indicates the acquired signal. This has shown the ideal case of applying a sinusoidal magnetic field.

We stored a serial of FTWs whose mapping in the MW source is multiple frequencies arranged at equal intervals on the spectrum in the slave FPGA RAM. During practical experimentation, we set the frequency sweeping range at 2.8 GHz to 2.95 GHz, with a step of 400 kHz between two frequencies. Consequently, this yields a total of 376 frequencies during the sweeping process. Additionally, the output microwave duration at each frequency is 30 ms, with a power level of approximately 17 dBm. The signal collected by the sampling module is normalized by the PC terminal, and the results are presented in Figure 7a.

As the NV centers have four kinds of different orientations, there are eight main resonances that exist on the spectrum of NV centers which can be divided into four groups which are marked with letters. Additionally, each resonance consists of two secondary resonances resulting from the hyperfine interactions caused by the ^14^N existing in the diamond [30]. We shall focus on the left resonance I marked in Figure 7a. When there is only an external static magnetic field, zero-field splitting *D* exists between sub-levels of $|m_{s}=\pm 1\rangle$ and $|m_{s}=0\rangle$ [31] and the value of *D* is 2.87 GHz and *D* is related to temperature. In a multipoint lock-in detection experiment, we apply AWG to generate a sinusoidal alternating current (AC) in copper coil, thereby generating an oscillating magnetic field in a fixed direction around the diamond. When external magnetic change occurs, resonances in the same group will create symmetrical movement, taking *D* as a baseline.

### 3.2. Multipoint Lock-in Detection

**Figure 8:**
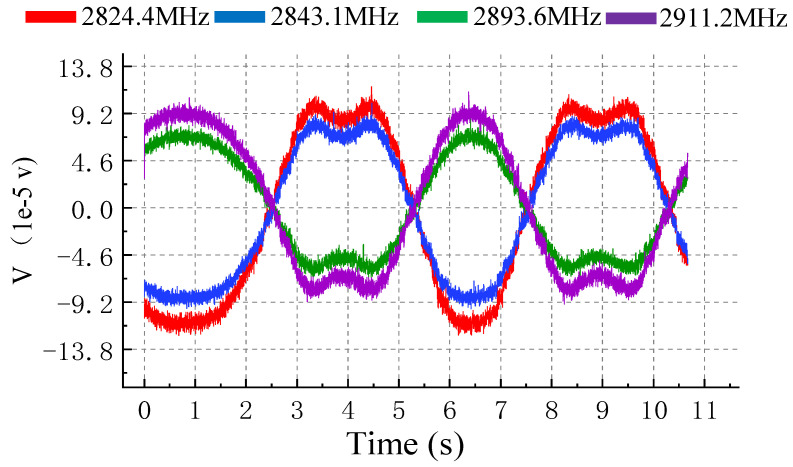
Result of multipoint lock-in detection. Four resonances in groups of I and II are detected step by step in an extremely short period of time. Frequency of fluorescence signal is correspond with external magnetic field whose frequency is 0.2 Hz.

**Figure 9:**
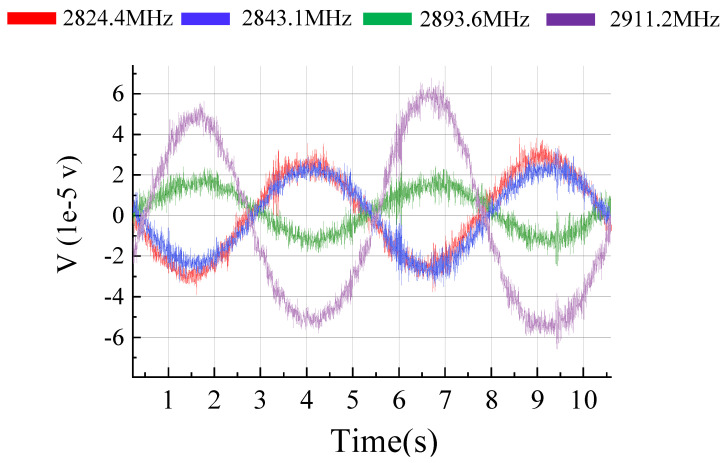
Result of multipoint lock-in detection. We set the parameters of alternating current on copper wire at 2.5 V, 0.2 Hz. Two pairs of resonance peaks were detected.

The primary function of the digital system we are presenting is the detection of multiple resonances using a single MW source. To validate the performance of the system, we conducted the following experiment: we configured FTWs in RAM whose mapping in the spectrum corresponds with frequencies of resonances according to the cw-ODMR experiment. During the actual operation, our focus was on two pairs of resonances, denoted as I and II in Figure 7a. It becomes apparent that differential operations are necessary to detect oscillating signals [32]. Utilizing the sampling method we reported in the section above, we can set Δf by adjusting FOW and classify the collected signal into section A and section B according to the level of trigger. Δf in Figure 7b represents the frequency difference between detection frequencies and center frequencies determined by FTW, and it is typically set to 100 kHz to 1000 kHz. Actual Δf is 500 kHz and we set the parameters of alternating current on copper wire at 5 V, 0.2 Hz. Fluorescence oscillation caused by the external magnetic field is shown in Figure 8. Two signals in the same group have inverse phase, which is consistent with the expected result. Note that the peak of the fluorescence signal has a depression; this occurrence is attributed to the significant shift of the resonance on the spectrum, which causes the main peak of the signal to extend beyond Δf . As a result, the signal from the secondary peak is collected by the DAQ system. It is worth noting that compared to Figure 8, the signal amplitude in this Figure 9 is smaller, and this is due to variations in the distance the resonance peaks move across the spectrum. Acquired signals are sinusoidal signals that correspond to the period of the external magnetic field. According to the above results, the performance of this method has been verified.

## 4. Conclusions

In this work, we report a digital system which is used for the detection of quantum magnetic-resonance. The system mainly integrates two FPGAs, including master and slave, and a DDS. Through the collaborative work between DAQ and MW source, the system can detect multiple resonances of NV centers with only one self-built MW source. Its performance has been proven by cw-ODMR and multiple experiments. Compared with traditional schemes that require the usage of multiple microwave sources, this system greatly reduces power consumption and complexity and because of its ability to detect magnetic-resonance of different NV orientations, it has potential applications in detecting the direction of magnetic fields and provides new ideas for manufacturing magnetic vector detection equipment. Since there is a time difference to the order of microseconds in the detection window for each frequency point, there is a certain phase difference between the data of each channel when outputting the magnetic signal data of multiple channels. When applied to low-frequency signal detection, the time difference is much larger than the microsecond time difference because the signal change period is to the order of seconds. However, when measuring high-frequency signals, the time difference between individual channels becomes noticeable due to the shortening of the change period of the signal to be measured, which is a point worthy of subsequent improvement. Additionally, if we can accelerate the clock main frequency of two FPGAs, the application of the system will no longer be limited to the detection of low-frequency magnetic fields.

## Data Availability

Data are contained within the article.

## References

[1] Barry J.F., Schloss J.M., Bauch E., Turner M.J., Hart C.A., Pham L.M., Walsworth R.L. (2020). Sensitivity optimization for nv-diamond magnetometry. Rev. Mod. Phys..

[2] Zhang J., Hegde S.S., Suter D. (2020). Efficient implementation of a quantum algorithm in a single nitrogen-vacancy center of diamond. Phys. Rev. Lett..

[3] Neumann P., Mizuochi N., Rempp F., Hemmer P., Watanabe H., Yamasaki S., Gaebel T., Jelezko F., Wrachtrup J. (2008). Multipartite entanglement among single spins in diamond. Science.

[4] Shi F., Zhang Q., Naydenov B., Jelezko F., Du J., Reinhard F., Wrachtrup J. (2013). Quantum logic readout and cooling of a single dark electron spin. Phys. Rev. B.

[5] Kong F., Ju C., Liu Y., Lei C., Wang M., Kong X., Wang P., Huang P., Li Z., Shi F. (2016). Direct measurement of topological numbers with spins in diamond. Phys. Rev. Lett..

[6] Wang Y., Dolde F., Biamonte J., Babbush R., Bergholm V., Yang S., Jakobi I., Aspuru-Guzik A., Whitfield J.D., Whitfield J.D. (2015). Quantum simulation of helium hydride cation in a solid-state spin register. ACS Nano.

[7] Maze J.R., Stanwix P.L., Hodges J.S., Hong S., Taylor J.M., Cappellaro P., Dutt M.V.G., Togan E., Zibrov A.S., Yacoby A. (2008). Nanoscale magnetic sensing with an individual electronic spin in diamond. Nature.

[8] Hall L.T., Beart G.C.G., Thomas E.A., Simpson D.A., McGuinness L.P., Manton J.H., Scholten R.E., Jelezko F., Wrachtrup J., Hollenberg L.C.L. (2012). High spatial and temporal resolution wide-field imaging of neuron activity using quantum nv-diamond. Sci. Rep..

[9] Jakobi I., Neumann P., Wang Y., Dasari D.B.R., El Hallak F., Bashir M.A., Edmonds A., Twitchen D., Wrachtrup J. (2017). Measuring broadband magnetic fields on the nanoscale using a hybrid quantum register. Nat. Nanotechnol..

[10] Shim J.H., Lee S.-J., Ghimire S., Hwang J.I., Lee K.-G., Kim K., Turner M.J., Hart C.A., Walsworth R.L., Oh S. (2022). Multiplexed sensing of magnetic field and temperature in real time using a nitrogen-vacancy ensemble in diamond. Phys. Rev. Appl..

[11] Schoenfeld R.S., Harneit W. (2011). Real time magnetic field sensing and imaging using a single spin in diamond. Phys. Rev. Lett..

[12] Dolde F., Fedder H., Doherty M.W., Nöbauer T., Rempp F., Wolf T., Reinhard F., Hollenberg L.C.L., Jelezko F. (2011). Electric-field sensing using single diamond spins. Nat. Phys..

[13] Li R., Kong F., Zhao P., Cheng Z., Qin Z., Wang M., Zhang Q., Wang P., Wang Y., Du J. (2020). Nanoscale electrometry based on a magnetic-field-resistant spin sensor. Phys. Rev. Lett..

[14] Kucsko G., Maurer P.C., Yao N.Y., Kubo M., Noh H.J., Lo P.K., Park H., Lukin M.D. (2013). Nanometre-scale thermometry in a living cell. Nature.

[15] Neumann P., Jakobi I., Dolde F., Burk C., Reuter R., Waldherr G., Honert J., Wolf T., Brunner A., Shim J.H. (2013). High-precision nanoscale temperature sensing using single defects in diamond. Nano Lett..

[16] Doherty M.W., Struzhkin V.V., Simpson D.A., McGuinness L.P., Meng Y., Karle T.J., Hemley R.J., Manson N.B., Hollenberg L.C., Hollenberg L.C.L. (2014). Electronic properties and metrology applications of the diamond nv- center under pressure. Phys. Rev. Lett..

[17] Barry J.F., Turner M.J., Schloss J.M., Glenn D.R., Song Y., Lukin M.D., Walsworth R.L. (2016). Optical magnetic detection of single-neuron action potentials using quantum defects in diamond. Proc. Natl. Acad. Sci. USA.

[18] Stimpson G., Skilbeck M., Patel R., Green B.L., Morley G. (2019). An open-source high-frequency lock-in amplifier. Rev. Sci. Instrum..

[19] Schloss J.M., Barry J.F., Turner M.J., Walsworth R.L. (2018). Simultaneous broadband vector magnetometry using solid-state spins. Phys. Rev. Appl..

[20] Santarini M. (2010). Xilinx redefines state of the art with new 7 series fpgas. Xcell J..

[21] Tierney J., Rader C., Gold B. (1971). A digital frequency synthesizer. IEEE Trans. Audio Electroacoust..

[22] Arablu M., Kafashi S., Smith S.T. (2018). Synchronous radio-frequency fm signal generator using direct digital synthesizers. Rev. Sci. Instrum..

[23] Qin X., Shi Z., Xie Y., Wang L., Rong X., Jia W., Zhang W., Du J. (2017). An integrated device with high performance multi-function generators and time-to-digital convertors. Rev. Sci. Instrum..

[24] Przybus B. (2010). Xilinx Redefines Power, Performance, and Design Productivity with Three New 28 nm Fpga Families: Virtex-7, Kintex-7, and Artix-7 Devices. Xilinx White Paper.

[25] Vindhya K., Bharathi B., Shenoy N.R., Kamala C. (2017). Performance evaluation of wide bandwidth rf signal generator chip. Int. J. Adv. Eng. Res. Sci..

[26] Anand N., Joseph G., Oommen S.S., Dhanabal R. (2014). Design and implementation of a high speed serial peripheral interface. Proceedings of the 2014 International Conference on Advances in Electrical Engineering (ICAEE).

[27] Liu Y., Ye R., Hu Q., Chen B., Zhang W., Zhou F., Xu N. (2023). A dds-based integrated microwave source for fast frequency sweeping in quantum magnetic-resonance systems. AIP Adv..

[28] Steiner M., Neumann P., Beck J., Jelezko F., Wrachtrup J. (2010). Universal enhancement of the optical readout fidelity of single electron spins at nitrogen-vacancy centers in diamond. Phys. Rev. B.

[29] Tetienne J., Rondin L., Spinicelli P., Chipaux M., Debuisschert T., Roch J., Jacques V. (2012). Magnetic-field-dependent photodynamics of single nv defects in diamond: An application to qualitative all-optical magnetic imaging. New J. Phys..

[30] Wang N., Liu C.-F., Fan J.-W., Feng X., Leong W.-H., Finkler A., Denisenko A., Li Q., Liu R.-B. (2022). Zero-field magnetometry using hyperfine-biased nitrogen-vacancy centers near diamond surfaces. Phys. Rev. Res..

[31] Kitazawa S., Matsuzaki Y., Saijo S., Kakuyanagi K., Saito S., Ishi-Hayase J. (2017). Vector-magnetic-field sensing via multifrequency control of nitrogen-vacancy centers in diamond. Phys. Rev. A.

[32] Ambal K., McMichael R.D. (2019). A differential rate meter for real-time peak tracking in optically detected magnetic resonance at low photon count rates. Rev. Sci. Instrum..

